# Obesity status and obesity-associated gut dysbiosis effects on hypothalamic structural covariance

**DOI:** 10.1038/s41366-021-00953-9

**Published:** 2021-09-01

**Authors:** O. Contreras-Rodriguez, M. Arnoriaga-Rodríguez, R. Miranda-Olivos, G. Blasco, C. Biarnés, J. Puig, J. Rivera-Pinto, M. L. Calle, V. Pérez-Brocal, A. Moya, C. Coll, L. Ramió-Torrentà, C. Soriano-Mas, J. M. Fernandez-Real

**Affiliations:** 1 Department of Psychiatry, Bellvitge University Hospital-IDIBELL, and CIBERSam-17 and CIBERObn (CB06/03/0034), Barcelona, Spain; 2 Department of Radiology-Medical Imaging (IDI), Girona Biomedical Research Institute (IdIBGi), Josep Trueta University Hospital, Girona, Spain; 3 Department of Psychiatry and Legal Medicine, Universitat Autònoma de Barcelona, Barcelona, Spain; 4 Health Institute Carlos III (ISCIII), Barcelona, Spain; 5 Department of Diabetes, Endocrinology and Nutrition-UDEN, and CIBERObn (CB06/03/0010), Girona, Spain; 6 Department of Medical Sciences, School of Medicine, University of Girona, Girona, Spain; 7 IrsiCaixa AIDS Research Institute, Badalona, Spain; 8 Biosciences Department, Faculty of Sciences and Technology, University of Vic-Central University of Catalonia, VIC Badalona, Spain; 9 Department of Genomics and Health, Foundation for the Promotion of Health and Biomedical Research of Valencia Region (FISABIO-Public Health), Valencia, Spain, and CIBEResp- CB06/02/0050, Madrid, Spain; 10 Institute for Integrative Systems Biology (I2SysBio), The University of Valencia and The Spanish National Research Council (CSIC-UVEG), Valencia, Spain; 11 Neuroimmunology and Multiple Sclerosis Unit, Department of Neurology, Girona Biomedical Research Institute (IdIBGi), Dr. Josep Trueta University Hospital, Girona, Spain; 12 Department of Psychobiology and Methodology of Health Sciences, Universitat Autònoma de Barcelona, Barcelona, Spain

**Keywords:** Hypothalamus, Obesity

## Abstract

**Background:**

Functional connectivity alterations in the lateral and medial hypothalamic networks have been associated with the development and maintenance of obesity, but the possible impact on the structural properties of these networks remains largely unexplored. Also, obesity-related gut dysbiosis may delineate specific hypothalamic alterations within obese conditions. We aim to assess the effects of obesity, and obesity and gut-dysbiosis on the structural covariance differences in hypothalamic networks, executive functioning, and depressive symptoms.

**Methods:**

Medial (MH) and lateral (LH) hypothalamic structural covariance alterations were identified in 57 subjects with obesity compared to 47 subjects without obesity. Gut dysbiosis in the subjects with obesity was defined by the presence of high (*n* = 28) and low (*n* = 29) values in a BMI-associated microbial signature, and posthoc comparisons between these groups were used as a proxy to explore the role of obesity-related gut dysbiosis on the hypothalamic measurements, executive function, and depressive symptoms.

**Results:**

Structural covariance alterations between the MH and the striatum, lateral prefrontal, cingulate, insula, and temporal cortices are congruent with previously functional connectivity disruptions in obesity conditions. MH structural covariance decreases encompassed postcentral parietal cortices in the subjects with obesity and gut-dysbiosis, but increases with subcortical nuclei involved in the coding food-related hedonic information in the subjects with obesity without gut-dysbiosis. Alterations for the structural covariance of the LH in the subjects with obesity and gut-dysbiosis encompassed increases with frontolimbic networks, but decreases with the lateral orbitofrontal cortex in the subjects with obesity without gut-dysbiosis. Subjects with obesity and gut dysbiosis showed higher executive dysfunction and depressive symptoms.

**Conclusions:**

Obesity-related gut dysbiosis is linked to specific structural covariance alterations in hypothalamic networks relevant to the integration of somatic-visceral information, and emotion regulation.

## Introduction

The role of the hypothalamus in energy regulation and the development and maintenance of obesity has been long acknowledged. Classical lesion studies led to the definition of the lateral hypothalamus as the feeding center and the ventromedial hypothalamus as the satiety center [1]. Current neurobiological accounts of obesity have however gained complexity, incorporating notions such as the impact that the rewarding and hedonic aspects of food and executive functions may have on eating behaviors [2–5]. Despite research on this topic has been scarce, most consistent findings indicate functional connectivity increases in people with obesity between the hypothalamus and the nucleus accumbens, orbitofrontal and temporal cortices, as well as decreases in functional connectivity with frontoparietal cortices [6–9].

Importantly, however, although resting-state functional connectivity assessments are perfectly suited to identify and characterize networks of connected regions, such estimations are limited to data gathered during the few minutes of data acquisition, therefore exclusively reflecting “current state” of individuals but not their recent history of connectivity across distant brain regions. Notwithstanding this, enduring functional connectivity across brain regions has been suggested to induce networks of structural covariance; that is, volume correlations across distant regions connected at the functional level [10, 11]. Therefore, assessment of structural covariance may relevantly inform about continuing hypothalamic network dysfunction in individuals with obesity. Nevertheless, despite preliminary evidence of hypothalamic structural alterations in obesity [12, 13], alterations in hypothalamic structural covariance patterns remain to be explored.

Beyond obesity, hypothalamic networks are also modulated by gut microbiota through different bidirectional and interlinked channels which supports the effect of obesity-related dysbiosis on eating behaviors [14, 15]. First, it has been shown that some bacterial strains influence the secretion of multiple hypothalamic hormones and peptides, as well as the production of neuroactive metabolites [15]. Particularly, the administration of specific bacterial strains altered the concentration of certain metabolites in the frontal cortex and the amygdala, and the gut microbiota affects brain dopamine levels crucially involved in food reward, addiction, and impulsive choice [15]. Also, obesity-like changes in the microbiota result in the permeabilization of the gut barrier and an increased passage of endotoxins, which may lead to low-grade peripheral endotoxemia and subsequent neuroinflammation in hypothalamic networks [16]. Finally, recent findings highlight the impact of the gut microbiota on structural brain development [17].

The present study aimed to assess structural covariance alterations linked to obesity in lateral and medial hypothalamic networks between subjects with and without obesity, as well as the potential modulating role of gut-dysbiosis on the hypothalamic alterations, executive functioning, and depressive symptoms of the subjects with obesity. We hypothesized that significant between-group differences in hypothalamic structural covariance networks will be congruent and complement those functional connectivity alterations described in previous studies [7, 8, 18–20]. Also, based on previous scientific evidences, we hypothesized gut-dysbiosis to have a relevant effect in explaining the structural covariance between the hypothalamus and other key brain regions involved in the regulation of eating behaviors (i.e., frontal cortices, amygdala, and nucleus accumbens) [14, 15, 17], executive functions and the presence of depressive symptoms [21–23] in those participants with obesity.

## Materials

### Subjects

One-hundred and four individuals participated in this cross-sectional study, 47 were non-obese (BMI range 19.20–29.90) and 57 had obesity (BMI range 30-58.60). The sample size is appropriate to study the hypothalamic networks based on previous studies with excess weight samples [7, 8]. Participants were recruited via the Endocrinology Department of Dr. Josep Trueta University Hospital. Eligible participants could be from both sexes, older than 18 years old and healthy, except for the presence of obesity. Exclusion criteria were: (i) presence of current or past medical illness (e.g., diabetes mellitus or impaired glucose tolerance, cancer, inflammatory-related illnesses) or incapacitating psychiatric disorders (e.g., major eating or psychiatric disorders, including eating disorders), as evidenced by semi-structured interviews, (ii) MRI contraindications (e.g., claustrophobia, ferromagnetic implants), (iii) excessive acute or chronic alcohol intake (i.e., ≥40 g OH/day in women or ≥80 g OH/day in men), (iv) clinical symptoms and signs of infection in the previous month or antibiotic, antifungal or antiviral treatment in the previous 3 months, and (v) pregnancy and lactation. Detailed characteristics of the study participants are provided in Table 1. The study data is available in https://thinkgut.eu/equipo/. The Institutional Review Board-Ethics Committee and the Committee for Clinical Research at the University Hospital of Girona Dr Josep Trueta (Girona, Spain) approved the study protocol. All procedures were in accordance with the ethical standards of the responsible committee on human experimentation (institutional and national) and with the Helsinki Declaration of 1975, as revised in 2008. All participants provided informed written informed consent prior to the start of the study.

**Table 1:** Demographic and health information of the 104 study participants with and without obesity.

Sample characteristics	Without-obesity (*n* = 47)	With obesity (*n* = 57)	Statistic
Main demographic variables			
Age (years)	49.65 ± 10.73	45.16 ± 10.12	0.030*
Sex (women/men)	30(63.8%)/17(36.2%)	39(68.4%)/18(31.6%)	0.387
Education (years)^a^	15.11 ± 2.78	12.06 ± 3.66	<0.001*
Education level^a^			
Elementary (*n* = 10)	90%	10%	
Secondary (*n* = 46)	73.91%	26.09%	
Higher (*n* = 44)	25%	75%	
Health/cognitive status			
BMI (kg/m^2^)	24.85 ± 2.67	42.75 ± 6.74	<0.001*
Fasting plasma glucose (mg/dL)	94.47 ± 13.6	96.28 ± 10.59	0.447
Glycated hemoglobin (%)	5.44 ± 0.27	5.54 ± 0.32	0.127
Cholesterol (mg/dL)	201.19 ± 35.59	192.60 ± 42.81	0.275
Triglycerides (mg/dL)	87.68 ± 41.38	120.56 ± 55.22	<0.001*
Fat mass (%)^b^	31.79 ± 7.38	50.12 ± 5.34	<0.001*
M venous (mg/[kg × min])^c^	9.43 ± 3.62	4.62 ± 2.56	<0.001*
Smoking (yes/no)	2(4.3%)/45(95.7%)	10(17.5%)/47(82.46%)	0.099
Alcohol intake (g/d)^d^	6.73 ± 9.08	1.62 ± 2.76	<0.001*
Stroop Interference^a^	46.13 ± 9.66	41.25 ± 9.56	0.013*
Depressive symptoms^a^	4.72 ± 3.69	7.57 ± 4.79	0.001*
Mean GM volume (ml)	702.37 ± 73.33	682.82 ± 57.72	0.131
Total Intracranial volume (ml)	1406.83 ± 140.79	1458.13 ± 169.66	0.103

Mean ± standard deviations are provided, except for sex, education level and smoking status where sample sizes and percentages are provided for women and men.

*p < 0.05

^a^Provided for *n* = 100/104 subjects,

^b^Fat mass is provided for *n* = 101/104 subjects,

^c^M venous is provided for *n* = 100/104 subjects,

^d^Alcohol intake is provided for *n* = 95/104 subjects.

### Measures

#### Brain imaging data acquisition and preprocessing

All participants were assessed on a 1.5-T Ingenia system (Philips Healthcare, Best, the Netherlands) with eight-channel head coils. Participants underwent a T1’ anatomical scan (TR = 8.3 ms, TE = 4.1 ms, flip angle = 8°, FOV = 230 × 190 mm, 232 × 229 pixel matrix; slice thickness = 1 mm). Data were processed and analyzed using MATLAB version R2017a (The MathWorks Inc, Natick, Mass) and Statistical Parametric Mapping software (SPM12; The Welcome Department of Imaging Neuroscience, London). Firstly, images were examined by an expert neuroradiologist to detect gross and clinically relevant anatomical abnormalities. Next, images were preprocessed using a standard procedure including three main steps: tissue segmentation, normalization to Montreal Neurological Institute (MNI) space, and smoothing. Images were segmented using the “new segment” algorithm, and the rigidly transformed versions of grey matter (GM) images derived from this algorithm were normalized using a Diffeomorphic Anatomical Registration Through Exponentiated Lie algebra algorithm (DARTEL) [24]. Specifically, using the option “create templates,” images were iteratively matched to a template generated from their own average, so as to generate a series of templates with increasing resolution. Native space GM images from participants were then registered to the highest resolution GM template within a high-dimensional diffeomorphic framework. Subsequently, spatially normalized tissue maps were modulated by the Jacobian determinants from the corresponding flow-fields to restore the volumetric information lost during the high-dimensional spatial registration. Normalized images were registered to the standard SPM template and re-sliced to a 2-mm resolution. Finally, images were smoothed with an 8-mm full-width at half-maximum isotropic Gaussian kernel.

#### Cognitive assessment

The Stroop Color-Word Test (Golden’s version [25]) was administered to assess executive functions. This version consists in three different parts: (1) reading words (color names) printed in black ink, (2) identification of the color ink of some signs (XXX printed in green, blue and red), and (3) identification of the color ink of incongruent color-word stimulus (interference effect). The subject is given 45 seconds for each task. We focused our interest in the interference effect score that imaging studies has consistently associated with prefrontal and parietal cortices [26, 27]. Depressive traits were assessed using the Patient Health Questionnaire 9 (PHQ-9), a module of the PRIME-D diagnostic instrument for mental disorders [28]. It encompasses nine items that range from 0 to 27. Scores if 5, 10, 15, and 20 represent cut-points for mild, moderate, moderately severe, and severe depressive symptoms, respectively.

#### Gut Microbiome

Stools samples from the study participants were obtained and preserved at –80 °C. Total DNA was extracted from frozen human stools using the QIAampDNAmini stool kit (Qiagen, Courtaboeuf, France), slightly modified by adding a bead (≤106 µm diameter) beating step (6500 rpm, 3 × 30 seconds). The region V3-V4 region of the bacterial 16 S rRNA gene was targeted by the primers 16S Amplicon PCR Forward Primer = 5′- tcgtcggcagcgtcagatgtgtataagagacagcctacgggnggcwgcag- 3′ and 16S Amplicon PCR Reverse Primer = 5′- gtctcgtgggctcggagatgtgtataagagacaggactachvgggtatctaatcc-3′, and sequenced by the MiSeq Desktop Sequencer (Illumina, San Diego, California, USA) using a MiSeq v3 Reagent Kit (Illumina). An average of 5000 sequences was generated.

### Analyses

#### Hypothalamic-based structural Covariance Analyses

Following prior work [6, 8, 29], lateral and medial hypothalamic subregions were identified in each hemisphere and respective seeds of interest were placed in the medial (MH *x* = ±4, *y* = 2, *z* = −12) and lateral (LH *x* = ±6, *y* = −10, *z* = −10) hypothalamus using 2-mm-radius spheres. The MH seed included the arcuate nucleus, as well as the ventromedial and parts of the dorsomedial hypothalamus. The central voxel of the LH seed was in the most posterior part of the region to minimize overlap with the MH seed and obtain maximally specific structural connectivity maps. Importantly, these seeds were spatially separated by more than 8 mm (>1 FWHM).

To calculate the whole-brain structural covariance patterns of our seeds of interest (MH, LH), we estimated 2 SPM models, 1 for each seed region. In all these analyses, we include only those voxels with a probability of being GM > 0.2. In addition, within each SPM model, variables of interest and covariables were sequentially orthogonalized following an iterative Gram-Schmidt procedure. Specifically, age was always the first to variable be entered, followed by sex, global grey matter volumes (GMV) (sum of all modulated voxel values), the other hypothalamic seed from the same hemisphere (e.g., for the LH the controlling seed was the MH seed), and finally, the hypothalamic seed of interest. Following such an approach, we aimed to remove from the seed of interest all the variance shared with the other hypothalamic region as well as with general confounding factors, therefore avoiding the inclusion of multiple collinear measurements in the design matrix. We then generated contrast images (beta values) to create *t* statistic maps of the within-group voxel-wise correlations (positive and negative) and the between-group correlation differences (obese vs. non-obese) in the patterns of structural covariance of the seed regions of interest (MH or LH) with the rest of the brain by using independent samples t-test with the seed of interest in interaction with group factor. The different confounding covariates were also included in this statistical model (see summary of main steps in the flow-chart of Figure S1). To correct for multiple comparisons (significant threshold set at *p* < 0.05, Family- Wise Error (FWE) corrected), voxel-wise non-parametric permutation testing [30] with 5000 permutations was performed using the Threshold-Free Cluster Enhancement (TFCE) technique [31] as implemented in the SPM-TFCE toolbox v117 (http://dbm.neuro.uni-jena.de/tfce/).

#### BMI-associated microbial signature effects on hypothalamic networks and Cognition in obese subjects

We apply *selbal* to search for a microbial signature that is predictive of BMI levels considering all the study participants [32]. We discarded those phyla with >20% of zeros. This resulted in 31 phyla (Supplemental Information S1) which were analyzed with *selbal* (code availability: repository “UVic-omics/selbal”). The aim of *selbal* is the identification of microbial signatures that are predictive of a phenotype of interest. Unlike other biomarker signatures that are defined as a linear combination of individuals markers, *selbal* microbial signatures have the form of what is called a “balance” in the compositional data analysis literature: two groups of taxa, group A and group B, whose relative abundance or balance is associated with the outcome of interest. Mathematically, the balance between A and B is defined as the log-transformed relative abundance between group A and group B. Positive values of the balance indicate that taxa in group A are more abundant than taxa B, and vice versa for negative values. *Selbal* is a forward regression algorithm: it first identifies the pair of taxon whose balance is most associated with the response, and subsequently, the remaining variables are assessed for inclusion in the balance, either in A or in group B, and those that improve the prediction accuracy are added to the microbial signature (i.e., R2 values). The median value of the identified BMI-associated microbial signature in the participants with obesity allowed us to compare for structural covariance differences in the hypothalamic networks between those subjects with obesity and higher vs lower estimates in this signature (identified as gut-dysbiosis and not-gut-dysbiosis groups). When significant, we also determined whether differences exist in relation to the non-obese group. As in the analyses comparing the obese and non-obese groups, this was investigated by generating within and between-group t statistic maps of the pattern of structural covariance of the seed regions of interest (MH or LH) with the rest of the brain, although, in this case, using ANOVA models with the seed of interest in interaction with group factor (non-obese, obese without gut-dysbiosis, and obese with gut-dysbiosis). Same confounding covariates were also included in this statistical model. Small-volume correction procedures were used to assess for significant between-group differences by creating spheres (3-mm and 5-mm radius spheres for subcortical and cortical regions, respectively) centered on the brain regions showing structural covariance differences in the subjects non-obese and the subjects with obesity. Differences in executive functions and presence of depressive symptoms between the groups with obesity, with or without gut-dysbiosis were assessed using an independent sample *t*-test in SPSS. The study variables meet normal distribution and have a similar variance between the groups (all *p* < 0.05).

## Results

### Structural covariance of hypothalamic networks

**Fig. 1:**
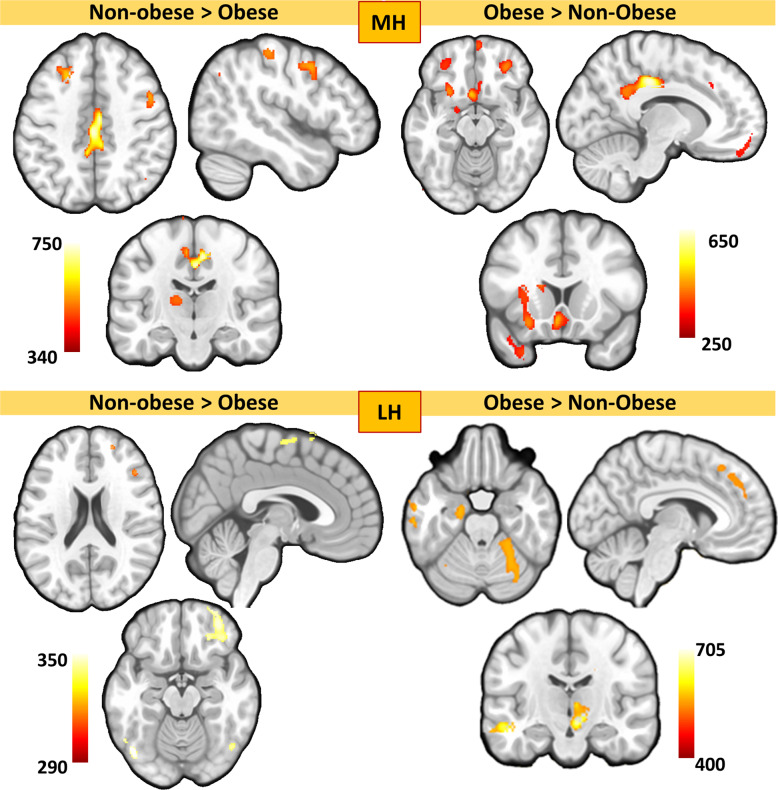
Differences in the structural covariance of the medial and lateral hypothalamic seed between the participants with and without obesity (*p* < 0.05 TFCE-FWE). The right hemisphere corresponds to the right side of axial and coronal views, and the right side of the sagittal lateral view. Color bars indicate TFCE values.

Between-group differences revealed an overall lower structural covariance in participants with obesity between the MH and the LH seeds and lateral prefrontal cortices, and sensorimotor processing regions (i.e., cortical motor areas, the ventrolateral thalamus). Participants with obesity also showed higher structural covariance between the MH and LH seeds and orbitofrontal and medial frontal areas (i.e., cingulate sections and the dorsomedial frontal cortices), the anterior insula, and several subcortical regions, such as the caudate, the amygdala, and the mediodorsal thalamus (Fig. 1, and Table 2). Within-group MH and LH structural covariance maps are provided in Supplemental Table S1. Results remained unchanged after controlling for fasting glucose levels and depressive symptoms.

**Table 2:** Differences in the whole-brain structural covariance patterns of medial (MH) and lateral (LH) hypothalamic seeds between the participants with and without obesity.

Seed	Associated region	MNI	t-TFCE	k_E_
		x, y, z	value	
MH LHL				
*Non-obese* *>* *Obese*	Middle Frontal gyrus	−26, 24, 47*	526.09	673
	Premotor cortex	47, 6, 45*	462.90	958
	Medial Primary motor cortex	0, -26, 41*	738.83	4775^a^
	Postcentral Parietal gyrus	45, −27, 56	440.98	4775^a^
		−42, −35, 56	380.13	959
	Ventrolateral thalamus	−14, −11, −3*	445.45	430
	Striate cortex	15, −105, 3	485.64	2827
*Obese* *>* *Non-obese*	Medial orbitofrontal cortex	2, 54, −29*	400.83	2571^a^
	Lateral orbitofrontal cortex	30, 41, −14*	370.52	2571^a^
		−32, 42, −14	290.16	231
	Subgenual cingulate cortex	−5, 14, −12*	442.21	5372^a^
	Dorsal cingulate cortex	−11, 29, 35	251.53	149
	Mid-posterior cingulate cortex	−11, −29, 39*	626.79	5372^a^
	Anterior insula	−27, 17, −14*	374.17	5372^a^
	Caudate	−12, 12, 8*	256.53	5372^a^
	Amygdala	−18, −8, −20	254.53	1587^a^
	Fusiform gyrus	−45, −59, −21*	393.11	3422
	Temporal pole	−30, 17, −36	304.64	1390
LH				
*Non-obese* *>* *Obese*	Superior frontal gyrus	21, 53, 20	323.78	90
	Inferior frontal gyrus	44, 30,18	328.67	132
	Lateral orbitofrontal cortex	35, 48, −15	340.32	657
	Pre-supplementary motor area Colliculi	2, −2, 66	307.55	337
	Lateral parietal cortex	−54, −39, 51	316.53	614
*Obese* *>* *Non-obese*	Dorsomedial frontal cortex	−6, 27, 50*	428.60	9183^a^
	Thalamus (mediodorsal, pulvinar)	9, −14, −9^	701.60	29531^a^
	Amygdala	−20, −8, −27*	461.74	29531^a^
	Middle temporal gyrus	−56, −24, −8*	588.82	29531^a^
	Cerebellum (VI-Cr I)	−35, −68, −39*	493.21	29531^a^

*kE* Cluster extent in voxels.

Coordinates (x, y, z) are given in Montreal Neurological Institute (MNI) atlas space. All results herein surpassed *p* < 0.05 TFCE FWE-corrections (*surpassed *p* < 0.01 TFCE FWE-corrections).

^a^part of the same cluster

Mean GM volume showed no significant differences between the subjects with and without obesity (Table 1). To further explore the link between BMI-related variations in hypothalamic structural covariance networks, we examined differences between the groups without obesity (BMI, *n* = 28), overweight (BMI ≤ 25–30, *n* = 19), and obesity (BMI > 30, *n* = 57) using linear contrasts in a second-level ANOVA model. Results are provided in Table S2. Finally, posthoc ANOVA models exploring interactions between sex (women, men) * obesity (with, without) indicate that sex did not have a major impact in the structural covariance of the MH and LH seeds (results not shown).

### BMI-associated microbial signature effects on Hypothalamic networks and Cognition in obese subjects

**Fig. 2:**
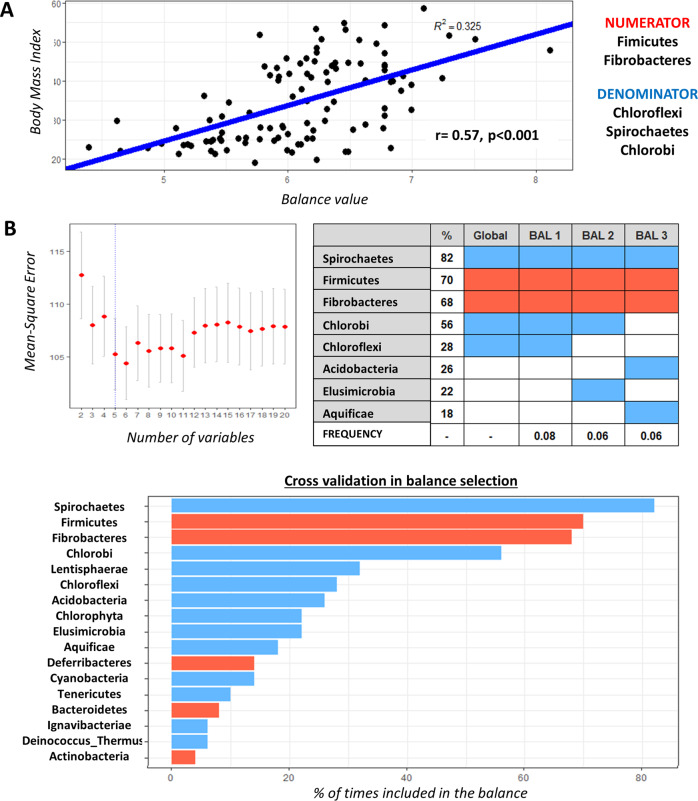
BMI-associated microbial signature. The BMI-associated microbial signature determined by *selbal* was given by the log-transformed relative abundances of {Firmicutes, Fibrobacteres} to {Chloroflexi, Chlorobi, Spirochaetes} (**A**). The cross-validation process determined five variables as the optimal number of phyla to be included in the balance as highlighted with a vertical dashed line (**B**, plot). The balance of phyla considered as the best BMI-associated microbial signature (global balance) coincides with the balance most frequently found in the cross-validation process, which turned to be the optimal balance 8% of the time (**B**, table). This was determined by exploring the balances obtained through the combination of eight of the most frequent phylums (**B**, bar graph). Particularly in the table the percentage of selection of each phylum in rows in given in the second column; the third column represents the global balance; and the last three columns represent other frequent balances (BAL 1–3). The last row indicates the proportion of times each balance was selected as optimal in the cross-validation procedure. Red and blue colored rectangles and bars indicate whether the phylum is in the numerator or the denominator position. White rectangles in the table indicate phylums not included in the balance.

*Selbal* determined that the balance of phyla most associated with BMI was given by the log-transformed relative abundances of {Firmicutes, Fibrobacteres} to {Chloroflexi, Chlorobi, Spirochaetes} (Fig. 2A). Mathematically, this relative abundances is obtained as $\log \left( {\frac{{gmean\left( {Firmicutes,Fibrobacteres} \right.}}{{gmean\left( {Chloroflexi,Chlorobi,Spirochaetes} \right)}}} \right)$, where *gmean* stands for geometric mean. Higher values in this signature were associated with higher BMI levels (*r* = 0.57) (mean ± SD, non-obese 5.72 ± 0.56 min = 4.39 and max = 6.82, obese 6.40 ± 0.53 min = 5.32 and max = 8.1). The robustness of the identified microbial signature can be evaluated in Fig. 2B. Within the subjects with obesity, those with a BMI gut-microbiota profile similar to the subjects without obesity were assigned to the non-gut-dysbiosis group (*n* = 29), while the rest made up the gut-dysbiosis group (*n* = 28) (according to a median value = 6.352) (groups information is provided in Table S3).

**Fig. 3:**
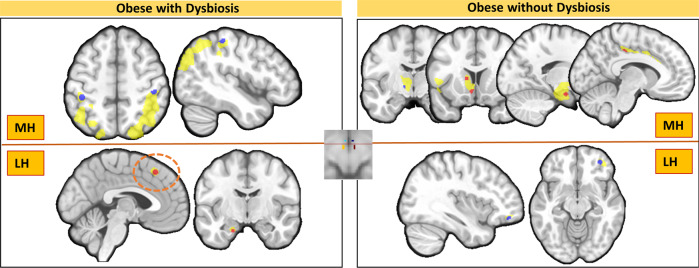
Medial (MH, top panels) and lateral (LH, bottom panels) hypothalamic networks showing a differential structural covariance between the subjects with obesity and gut-dysbiosis (left) vs those without gut-dysbiosis (right). Lower and higher between-group structural covariance differences are indicated in blue and red, respectively. Significant findings (*p*_SVC-FWE_ < 0.05) are shown on the subthreshold clusters (yellow) showing a tendency towards differences in the structural covariance of the MH and LH between those groups to provide a better context of the anatomy implicated. Circles indicate regions whose structural covariance with the hypothalamic seeds differs in both obese groups vs subjects without obesity.

For the MH seed, the subjects with obesity and gut-dysbiosis had lower structural covariance with the postcentral parietal cortices compared to the subjects with obesity without gut-dysbiosis, as well as the subjects without obesity. In turn, the subjects with obesity without gut-dysbiosis had lower structural covariance between the MH seed and the ventrolateral thalamus, and higher structural covariance with the left amygdala, dorsal caudate, and subgenual and middle posterior sections of the cingulate cortex compared to the subjects with obesity and gut-dysbiosis and the subjects without obesity (Table S4, Fig. 3).

For the LH seed, the subjects with obesity and gut-dysbiosis had higher structural covariance with the left amygdala, and the dorsomedial prefrontal cortex compared to the subjects with obesity without gut-dysbiosis and the subjects without obesity. Of note, the subjects with obesity without gut-dysbiosis also showed higher structural covariance in the LH-dorsomedial prefrontal cortex network compared with the subjects non-obese. In turn, the subjects with obesity without gut-dysbiosis had lower structural covariance with the right lateral orbitofrontal cortex compared to the subjects with obesity and gut-dysbiosis and the subjects non-obese (Table S4, Fig. 3). Mean GM volume showed no significant differences between the subjects with obesity with and without gut-dysbiosis (Table S3).

Descriptive statistics of the cognitive variables are provided in Tables 1 and 2 for each of the study groups. Subjects with obesity and gut-dysbiosis showed higher interference in the Stroop task (*p* = 0.002) and a greater presence of depressive symptoms (*p* < 0.001) compared with the subjects without obesity. No significant differences emerged when these variables were compared between the subjects with obesity without gut-dysbiosis and the subjects without obesity (all *p* < 0.05). Between the groups with obesity, those with gut-dysbiosis showed higher interference in the Stroop task (*p* = 0.032), although a tendency for a greater presence of depressive symptoms (*p* = 0.067), compared with those without gut-dysbiosis. The difference found for the interference in the Stroop remained even after controlling for the statistical differences in age between the subjects with obesity with and without gut-dysbiosis (*p* = 0.021).

## Discussion

The present findings provide, to our knowledge, the first evidence that previously reported functional connectivity disruptions in hypothalamic networks linked to obesity are also present in terms of its structural features. Furthermore, the obesity-associated hypothalamic structural covariance alterations were distinct in the subjects with obesity with and without gut-dysbiosis. Particularly, MH structural covariance alterations largely encompassed regions part of the postcentral parietal gyri in the gut-dysbiosis group, but subcortical nuclei in the non-gut-dysbiosis group. Discrete differences emerged for the structural covariance of the LH, which in the gut-dysbiosis group encompassed the basolateral amygdala and the dorsomedial prefrontal cortex, but the right lateral orbitofrontal cortex in the non-gut-dysbiosis group.

Higher structural covariance in the MH-caudate network in the subjects with vs without obesity herein is congruent with the previously reported higher functional connectivity in this network in similar samples [6, 8, 33], which has been linked to the ability to cut down food intake [34]. Also, fasting states [19, 33] had a differential impact on the functional connectivity between the MH and the putamen, and insular and middle cingulate cortices in subjects with obesity. The higher structural covariance with the temporal cortex is congruent with the increased functional connectivity in this network in adult [8] and adolescent [7] participants with excess weights, thought to be associated with retrain eating and BMI. Lower functional connectivity between the MH and lateral prefrontal cortices has also been a recurrent finding across studies [7, 8]. Nevertheless, the higher structural covariance in the LH-cerebellum network in the participants with obesity is unexpected as lower functional connectivity was previously reported in individuals with excess weight [7, 8]. However, the congruence of this finding with previous studies is limited as, to our knowledge, no study has characterized the functional connectivity of the LH during fasting states in samples with obesity.

Subjects with obesity and gut-dysbiosis showed the lowest structural covariance between the MH and postcentral parietal cortices. Satiation is associated with increased regional cerebral blow flow in these cortices [35], and enhanced functional connectivity in the hypothalamus-parietal cortex network [9], which implicates these areas in the suppression of the eating drive. Cumulating literature supports that individual differences in the structural properties of this network are associated with the inhibition of behavioral responses to food cues, with an ineffective function of these networks being associated with overconsumption in obesity [5]. In support, subjects with obesity and with dysbiosis showed the highest interference effect during the Stroop task, an effect known to be reliant on this parietal network [26, 27]. This finding is congruent with previous evidence linking the gut microbiota and the performance in the Stroop task [21]. These findings may indicate gut-dysbiosis to boost the risk to the obese phenotype through an inability to suppress eating behaviors after satiation [9, 36, 37]. Within the hypotheses set to explain the contribution of the gut microbiome to obesity [38], this interpretation may be congruent with preclinical evidence linking gut dysbiosis with hyperphagia after prolonged high-fat diets. It is also consistent with the modulation of satiation and obesity by probiotics and fermentable carbohydrates and fibers (ex. short-chain fatty acids and ß-glucans), effects though to be possible through activation of the dorsal vagal complex and main hypothalamic regions [39–42].

In addition, the subjects with obesity and gut-dysbiosis showed the highest structural covariance between the LH and the dorsomedial prefrontal cortex and the amygdala, key regions in the tuning of emotions [43, 44]. The LH itself has been suggested to play a predominant role in the regulation of both feeding and emotions (e.g., stress [45]), being particularly relevant in the preference for palatable food cues under negative emotional states [46, 47]. Several studies have shown that the gut microbiome influences emotional behaviors and the key underlying frontolimbic networks [22, 23]. Obese conditions are highly comorbid with the presence of depression and anxiety and shared gut microbiota mechanisms between these pathologies have been emphasized [48]. In support, subjects with obesity and dysbiosis showed the highest presence of depressive symptoms, although this was only significant when compared with the subjects without obesity.

The alteration in the hypothalamic structural covariance maps of subjects with obesity without gut-dysbiosis may be more consistent with neuro-computational perspectives that tackle feeding decisions through linking the effects of metabolic and endocrine factors on the decision-making circuitry [49]. In this line, the higher structural covariance of the MH and subcortical nuclei (i.e., the amygdala and caudate) may reflect a decreased effect of hormonal satiety signals (e.g., leptin, insulin) on the function of these regions [50, 51]. This is congruent with metabolic-associated changes in functional connectivity of this hypothalamic network after a meal in subjects without obesity, whereas these networks were enhanced during fasting and not affected by food intake in subjects with obesity [20]. Overall, these alterations have been associated with the likelihood of large hedonic responses or reward prediction errors after food consumption and impulsive food choices [49]. Furthermore, lower structural covariance between the LH and the lateral orbitofrontal cortex may also contribute to an inefficient regulation of feeding behavior through alterations in assigning correct motivational values to food cues [52, 53]. Finally, the additional changes in the structural covariance between the MH and the ventrolateral thalamus, and middle and subgenual sections of the cingulate cortex, implicated motor and autonomic aspects of behavior, may also contribute to impulsive and unhealthy feeding behaviors in these subjects under sated states [54, 55].

These findings need to be appraised in the context of the study’s limitations. First, the specific mechanisms whereby gut microbiota-induced obesity affects the morphological features of hypothalamic networks are not revealed in this present study [38]. In addition, our results are correlational and thus animal models and longitudinal studies are needed to examine the causal role of obesity and associated microbial signatures on the hypothalamic networks. Finally, future studies may investigate the link between the reported brain findings and eating behaviors relevant to obesity. Notwithstanding the limitations, our findings may have important implications for obesity treatment. On the one hand, they support the importance of considering the host microbiota to fully understand the neural substrate and cognition in obesity conditions. In addition, they provide new information on the neural substrates underlying obesity that can provide a basis for further testing-focused interventions.

BMI-associated gut dysbiosis may contribute to the obesity phenotype through specific alterations in medial hypothalamic-postcentral parietal networks, involved in the inhibition of behavioral responses to food cues in satiation, and concomitant problems in lateral hypothalamic-frontolimbic networks that may contribute to inadequate emotion regulation processes. The present study supports the importance of considering the host microbiota to fully understand the neural substrate in obesity conditions. New findings may provide a basis for further understanding the underlying gut-microbiota mechanisms in the context of obesity and testing-focused interventions.

## Supplementary information

Supplemental Information_final
