# Supplementary material for: Obesity status and obesity-associated gut dysbiosis effects on hypothalamic structural covariance
Source: Int J Obes (Lond). 2021 Sep 1;46(1):30–8. doi: 10.1038/s41366-021-00953-9 (PMC8748191; doi:10.1038/s41366-021-00953-9)
Supplement: Supplementary file 1 — Supplemental Information_final [file 41366_2021_953_MOESM1_ESM.doc]

**Supplemental Information**

**Obese hypothalamic structural covariance alterations are linked to BMI-gut dysbiosis**

O Contreras-Rodriguez, PhD^1^*, M Arnoriaga-Rodríguez, MSc*, R Miranda-Olivos, MSc, G Blasco, PhD, C Biarnés, MSc, J Puig, PhD, J [Rivera-Pinto](https://www.ncbi.nlm.nih.gov/pubmed/?term=Rivera-Pinto J%5BAuthor%5D&cauthor=true&cauthor_uid=30035234), PhD, ML Calle, PhD, V Pérez-Brocal, PhD, A Moya, PhD, C Coll, MSc, L Ramió-Torrentà, PhD, C Soriano-Mas, PhD, JM Fernandez-Real, PhD

**S1.**  List of phyla included in selbal to determine the microbial signatures predictive of BMI.

Acidobacteria

Actinobacteria

Aquificae

Armatimonadetes

Bacteroidetes

Candidatus_Omnitrophica

Candidatus_Saccharibacteria

Chlamydiae

Chlorobi

Chloroflexi

Chlorophyta

Cyanobacteria

Deferribacteres

Deinococcus_Thermus

Elusimicrobia

Euryarchaeota

Fibrobacteres

Firmicutes

Fusobacteria

Gemmatimonadetes

Ignavibacteriae

Lentisphaerae

Microsporidia

Nitrospirae

Planctomycetes

Proteobacteria

Spirochaetes

Synergistetes

Tenericutes

Thermotogae

Verrucomicrobia

**Figure S1**. Flowchart of the steps in the analysis of the structural covariance of the medial hypothalamus as a seed of interest. Abbreviations: MH, Medial Hypothalamus; LH, Lateral Hypothalamus; GMV, gray matter volume.


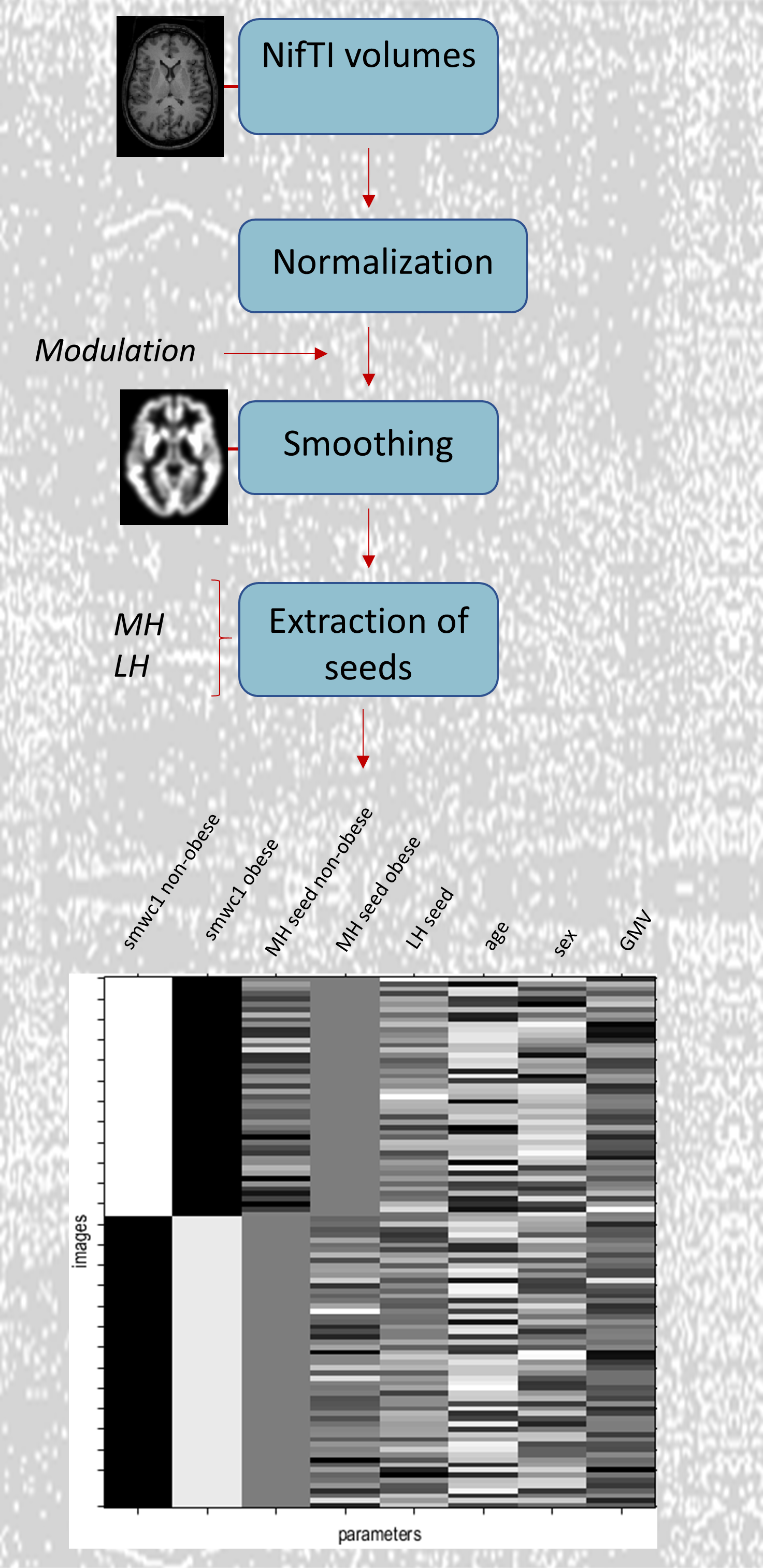


**Table S1**. Positive and negative structural covariance maps for the medial (MH) and the lateral (LH) hypothalamic seeds in the participants with and without obesity.

| **Seed** | **Connected region** | **Non-obese (n= 47)** | | **Obese (n= 57)** | | | |  | |
| --- | --- | --- | --- | --- | --- | --- | --- | --- | --- |
|  |  | **x, y, z** | **t-TFCE** | | **x,y,z** | **t-TFCE** |  | | |
| **MH** | *Positive connectivity* | | | | | |  | |  |
|  | Middle Frontal gyrus | 30, 29, 44 | 696.18 | |  | ns |  | | |
|  |  | -21, 24, 41 | 996.86 | |  | ns |  | | |
|  | Inferior Frontal gyrus | 38, 17, 27 | 631.73 | |  | ns |  | | |
|  |  | -36, 17, 27 | 809.98 | |  | ns |  | | |
|  | Caudate | 8, 6, 12 | 1933.5 | | 11, 11, 9 | 1227.23 |  | | |
|  |  | -9, 2, 12 | 1025.9 | | -11, 8, 11 | 2316.10 |  | | |
|  | Ventral Putamen |  | ns | | -26, -3, -2 | 1246.22 |  | | |
|  | Middle-Posterior Insula |  | ns | | 47, -6, 0 | 681.38 |  | | |
|  |  | -42, -2, 5 | 675.82 | | -36, -21, 11 | 657.32 |  | | |
|  | Medial Thalamus | 3, -20, 3 | 3578.9 | | 0, -17, 3 | 4273.15 |  | | |
|  | Somatosensory cortex | -54, -24, 38 | 638.20 | |  | ns |  | | |
|  | Temporal pole |  | ns | | -48, 8, -11 | 834.84 |  | | |
|  | Superior Temporal gyrus | -54, -9, 0 | 639.15 | |  | ns |  | | |
|  | Mid-Posterior Cingulate cortex |  | ns | | -9, -27, 42 | 809.88 |  | | |
|  | Dorsal Posterior Cingulate cortex |  | ns | | 2, -39, 38 | 745.00 |  | | |
|  | Cerebellum |  | ns | | 18, -42, -29 | 650.02 |  | | |
|  | *Negative connectivity* | | | | | |  | | |
|  | Ventral Thalamus |  | ns | | -11, -8, -6 | 2693.7 |  | | |
|  |  |  |  | |  |  |  | | |
| **LH** |  | *Positive connectivity* | | | |  |  | | |
|  | Middle Frontal gyrus | 24, 51, 24 | 691.49 | |  | ns |  | | |
|  |  | -36, 30, 29 | 745.50 | |  | ns |  | | |
|  | Dorsomedial prefrontal cortex | -15, 33, 47 | 691.09 | |  | ns |  | | |
|  | Orbitofrontal cortex | -9, 63, -12 | 790.09 | |  | ns |  | | |
|  | Premotor cortex | 44, -6, 41 | 878.16 | |  | ns |  | | |
|  | Pre-Supplementary motor cortex | 3, -8, 68 | 648.95 | |  | ns |  | | |
|  | Ventral Caudate | 12, 12, -2 | 593.63 | | 11, 9, -5 | 1282.24 |  | | |
|  |  | -15, 12, -2 | 1309.6 | | -11, 9, -5 | 1391.98 |  | | |
|  | Dorsal Putamen | 27, -5, 12 | 944.11 | |  | ns |  | | |
|  |  | -24, -3, 15 | 1140.5 | |  | ns |  | | |
|  | Midbrain | 2, -20, -14 | 2293.5 | | 2, -20, -14 | 4460.49 |  | | |
|  | | *Negative connectivity* | | | |  |  | | |
|  | |  | | | |  |  | | |
|  | Caudate | -11, 20, -3 | 1034.1 | |  | ns |  | | |
|  | Thalamus | 3, -5, 3 | 1318.9 | |  | ns |  | | |
|  |  | 12, -23, 5 | 1074.9 | |  | ns |  | | |
|  | Amygdala | -24, 3, -18 | 1285.0 | |  | ns |  | | |
|  | Middle Temporal gyrus | -54, -23, -9 | 1229.9 | |  | ns |  | | |
|  | Lingual gyrus | -21, -57, -9 | 1020.8 | |  | ns |  | | |
|  | Cerebellum | -2, -48, -8 | 1060.6 | |  | ns |  | | |
|  |  |  |  | |  |  |  | | |

Anatomical coordinates (x, y, z) are given in Montreal Neurological Institute (MNI) Atlas space. Abbreviations: ns, non-significant. All results herein surpassed p<0.05 TFCE FWE-corrections.

**Table S2**. Difference in whole-brain structural covariance patterns of medial (MH) and lateral (LH) hypothalamic seeds between the participants without obesity (BMI, 18.5-24.9), overweight (BMI, 25-29.9) or obesity (BMI<30).

| **Seed** | **Associated region** | **MNI coordinates** | | | | **t-TFCE** | | |
| --- | --- | --- | --- | --- | --- | --- | --- | --- |
|  |  | **x, y, z** | | | | **value** | | |
| **MH**  **LHL** |  | |  |  | | |  | |
|  |  | |  |  | | |  | |
| Obese>Controls>Overweight | **Subgenual Cingulate cortex** | -3, 14, -12 | | | | 357.76 | | |
|  |  |  | | | |  | | |
| Controls>Obese>Overweight | **Lateral Orbitofrontal cortex** | -33, 48, -8 | | | | 371.14 | | |
|  |  | 35, 50, -9 | | | | 318.70 | | |
|  |  |  | | | |  | | |
| Overweight> Obese> Controls | **Dorsal Cingulate cortex** | 3, 18, 36 | | | | 960.66 | | |
|  | **Anterior Insula** | 44, -2, 5 | | | | 493.74 | | |
|  |  |  | | | |  | | |
| Overweight>Controls>Obese | **Posterior Cingulate cortex** | 0, -39, 36 | | | | 912.66 | | |
|  | **Anterior Insula** | -42, 0, 5 | | | | 769.62 | | |
|  |  |  | | | |  | | |
| **LH** |  | |  | |  | | |  |
|  |  |  | | | |  | | |
| Obese>Controls>Overweight | **Middle Temporal cortex** | -54, -26, -6 | | | | 598.29 | | |
|  | **Inferior Frontal gyrus** | -57, 20, 17 | | | | 424.84 | | |
|  |  |  | | | |  | | |
| Controls>Overweight>Obese | **Lateral Orbitofrontal cortex** | -38, 35, -15 | | | | 263.78 | | |
|  |  |  | | | |  | | |
| Controls>Obese>Overweight | **Superior Frontal gyrus** | 18, 57, 21 | | | | 157.42 | | |
|  | **Cerebellum (Lobule IX)** | 12, -51, -44 | | | | 371.56 | | |
|  |  |  | | | |  | | |
| Overweight> Obese> Controls | **Anterior Insula** | -29, 17, -12 | | | | 537.94 | | |
|  | **Mid-anterior Cingulate cortex** | -6, -2, 36 | | | | 997.56 | | |
|  |  |  | | | |  | | |
| Overweight>Controls>Obese | **Subgenual Cingulate cortex** | 3, 17, -14 | | | | 423.48 | | |
|  |  |  | | | |  | | |

Coordinates (x, y, z) are given in Montreal Neurological Institute (MNI) atlas space. All results herein surpassed p<0.05 TFCE FWE-corrections.

Coordinates (x, y, z) are given in Montreal Neurological Institute (MNI) atlas space. All results herein surpassed p<0.05 TFCE FWE-corrections. k_E_, Cluster extent in voxels. * part of the same cluster.

**Table S3.** Demographic and health information of the participants with obesity with and without gut-dysbiosis as determined by the BMI-associated microbial signature.

| **Sample characteristics** | **No-dysbiosis**  **(n= 29)** | | **Dysbiosis**  **(n= 28)** | **Statistic** |
| --- | --- | --- | --- | --- |
| **Main demographic variables** | |  | | |
| Age (years) | 48.34 ± 9.12 | | 41.86 ± 10.20 | 0.014* |
| Sex (women/men) | 22(75.9%)/7(24.1%) | | 17(60.7%)/11(39.3%) | 0.219 |
| Education (years)^1^ | 12.55 ± 3.69 | | 11.48 ± 3.62 | 0.288 |
| **Health/Cognitive status** |  | |  |  |
| BMI (kg/m^2^) | 41.37 ± 6.17 | | 44.18 ± 7.11 | 0.116 |
| Fasting glucose (mg/dL) | 95.86 ± 10.88 | | 96.71 ± 10.46 | 0.764 |
| Glycated hemoglobin (%) | 5.51 ± 0.21 | | 5.56 ± 0.41 | 0.591 |
| Cholesterol (mg/dL) | 201.10 ± 47.54 | | 183.79 ± 36.03 | 0.128 |
| Triglycerides (mg/dL) | 125.86 ± 62.32 | | 115.07 ± 47.27 | 0.466 |
| Fat mass (%)^2^ | 50.02 ± 5.57 | | 50.24 ± 6.12 | 0.877 |
| M venous (mg / [kg × min])^3^ | 4.36 ± 2.35 | | 4.92 ± 2.80 | 0.443 |
| Smoking (yes/no) | 4(13.8%)/25(86.2%) | | 6(21.4%)/22(78.6%) | 0.699 |
| Alcohol intake (g/d)^2^ | 1.73 ± 3.02 | | 1.48 ± 2.48 | 0.744 |
| Stroop Interference^4^ | 43.89 ± 8.89 | | 38.28 ± 9.59 | 0.032* |
| Depressive symptoms^4^ | 6.43 ± 4.84 | | 8.84 ± 4.48 | 0.067 |
| Mean GM volume (ml) | 673.93 ± 50.14 | | 692.09 ± 64.26 | 0.240 |
| Total Intracranial volume (ml) | 1443.54 ± 169.74 | | 1472.22 ± 171.37 | 0.528 |

Mean ± standard deviations are provided, except for sex where sample sizes and percentages are provided for women and men. ^1^Provided for n= 54/57 subjects, ^2^Provided for n=55/57 subjects, ^3^M venous is provided for 51/57, ^4^Cognitive information is provided for 53/57 subjects with obesity. * p<0.05

**Table S4.** Structural covariance patterns differences on the medial (MH) and lateral (LH) hypothalamic seeds between the participants with and without BMI-gut associated dysbiosis.

| **Seed** | **Associated region** | **MNI coordinates** | | | | **t-** | | **p_SVC-FWE_** | |
| --- | --- | --- | --- | --- | --- | --- | --- | --- | --- |
|  |  | **x, y, z** | | | | **value** | |  | |
| **MH**  **LHL** |  | |  |  | | |  | |  |
| **Dysbiosis**<No-dysbiosis | **Postcentral gyrus R** | **44, -32, 56** | | | | **3.34** | | **0.011** | |
|  | *Dysbiosis<Non-obese* | *44, -29, 56* | | | | *4.25* | | *0.001* | |
|  | *No-dysbiosis vs Non-obese* |  | | | | *ns* | |  | |
|  | **Postcentral gyrus L** | **-44, -35, 53** | | | | **3.82** | | **0.004** | |
|  | *Dysbiosis<Non-obese* | *-42, -35, 54* | | | | *4.58* | | *0.001* | |
|  | *No-dysbiosis vs Non-obese* |  | | | | *ns* | |  | |
|  |  |  | | | |  | |  | |
|  |  |  | | | |  | |  | |
| **No-dysbiosis**<Dysbiosis | **Ventrolateral Thalamus** | **-12, -11, -2** | | | | **5.07** | | **<0.001** | |
|  | *No-dysbiosis<Non-obese* | *-12, -9, -3* | | | | *6.54* | | *<0.001* | |
|  | *Dysbiosis vs Non-obese* |  | | | | *ns* | |  | |
|  |  |  | | | |  | |  | |
| **No-dysbiosis**>Dysbiosis | **Caudate** | **-12, 14, 6** | | | | **2.47** | | **0.038** | |
|  | *No-dysbiosis>Non-obese* | *-12, 14, 6* | | | | *2.72* | | *0.022* | |
|  | *Dysbiosis vs Non-obese* |  | | | | *ns* | |  | |
|  | **Amygdala** | **-18, -9, -21** | | | | **3.98** | | **0.001** | |
|  | *No-dysbiosis>Non-obese* | *-18, -11, -20* | | | | *3.63* | | *0.002* | |
|  | *Dysbiosis vs Non-obese* |  | | | | *ns* | |  | |
|  | **Subgenual Cingulate cortex** | **-6, 12, -8** | | | | **2.91** | | **0.043** | |
|  | *No-dysbiosis>Non-obese* | *-5, 15, -12* | | | | *4.44* | | *0.001* | |
|  | *Dysbiosis vs Non-obese* |  | | | | *ns* | |  | |
|  | **Mid-posterior Cingulate cortex** | **-9, -27, 44** | | | | **3.18** | | **0.023** | |
|  | *No-dysbiosis>Non-obese* | *-8, -26, 41* | | | | *4.40* | | *0.001* | |
|  | *Dysbiosis vs Non-obese* |  | | | | ns | |  | |
| **LH** |  | |  | |  | | |  | |
| **Dysbiosis**>No-dysbiosis | **Amygdala** | **-20, -6, -26** | | | | **2.98** | | **0.012** | |
|  | *Dysbiosis>Non-obese* | *-20, -8, -26* | | | | *3.72* | | *0.001* | |
|  | *No-dysbiosis vs Non-obese* |  | | | | *ns* | |  | |
|  |  |  | | | |  | |  | |
|  | **Dorsomedial Frontal cortex**  Colliculi | **-5, 26, 51** | | | | **3.68** | | **0.004** | |
|  | *Dysbiosis>Non-obese* | *-6, 27, 50* | | | | *3.55* | | *0.006* | |
|  | *No-dysbiosis>Non-obese* | *-11, 26, 48* | | | | *3.18* | | *0.016* | |
|  |  |  | | | |  | |  | |
| **No-dysbiosis**<Dysbiosis | **Lateral Orbitofrontal cortex** | **38, 48, -12** | | | | **3.02** | | **0.033** | |
|  | *No-dysbiosis<Non-obese* | *35, 50, -14* | | | | *3.78* | | *0.004* | |
|  | *Dysbiosis vs Non-obese* |  | | | | *ns* | |  | |

Coordinates (x, y, z) are given in Montreal Neurological Institute (MNI) atlas space. All results herein surpassed p_SVC-FWE_ < 0.05 using small-volume correction procedures. MNI coordinates for each of the networks showing significant difference in the structural covariance with the seed of interest in the No-dysbiosis vs the Dysbiosis obese participants are indicated in bold. Ns= non-significant.
